# Sample Preparation for *in vitro* Analysis of Iodine in Thyroid Tissue using X-ray Fluorescence

**Published:** 2008-04-10

**Authors:** Marie Hansson, Mats Isaksson, Gertrud Berg

**Affiliations:** 1 Department of Radiation Physics, Göteborg University, Göteborg, Sweden; 2 Department of Oncology, Sahlgrenska University Hospital, Göteborg, Sweden

**Keywords:** thyroid, iodine, X-ray fluorescence, secondary ion mass spectrometry, fixation, sample preparation

## Abstract

Iodine is enriched and stored in the thyroid gland. Due to several factors, the size of the thyroid iodine pool varies both between individuals and within individuals over time. Excess iodine as well as iodine deficiency may promote thyroid cancer. Therefore, knowledge of iodine content and distribution within thyroid cancer tissue is of interest. X-ray fluorescence analysis (XRF) and secondary ion mass spectrometry (SIMS) are two methods that can be used to assess iodine content in thyroid tissue. With both techniques, choice of sample preparation affects the results. Aldehyde fixatives are required for SIMS analysis while a freezing method might be satisfactory for XRF analysis. The aims of the present study were primarily to evaluate a simple freezing technique for preserving samples for XRF analysis and also to use XRF to evaluate the efficacy of using aldehyde fixatives to prepare samples for SIMS analysis. Ten porcine thyroids were sectioned into four pieces that were either frozen or fixed in formaldehyde, glutaraldehyde, or a modified Karnovsky fixative. The frozen samples were assessed for iodine content with XRF after 1 and 2 months, and the fixed samples were analyzed for iodine content after 1 week. Freezing of untreated tissue yielded no significant iodine loss, whereas fixation with aldehydes yielded an iodine loss of 14–30%, with Karnovsky producing the least loss.

## Introduction

The rare element iodine is essential for production of the thyroid hormones thyroxin and triiodothyronine, which are important for maintaining normal body growth and metabolism. Iodine is present in low concentrations in the environment, chiefly as dissolved iodide in seawater. Iodine is available in variable amounts in food. In the thyroid, it can be enriched and stored in the intrathyroidal iodine pool. Although the thyroid can adapt to changes in iodine intake, both insufficient and excessive intake may lead to thyroid disease (Rousset and Dunn, 2007).

Studies have shown that iodine plays a role in the genesis and progression of thyroid cancer. In a rat model, iodine deficiency and also iodine excess promoted the development of thyroid cancer in the presence of a carcinogenic agent (Kanno et al. 1992). In other studies, iodine deficiency enhanced the progression of thyroid cancer in mice and rats (Fang et al. 1994; Ohshima and Ward, 1986). The mechanisms working in tumor promotion are unknown but several suggestions and hypotheses are presented; some of them speculating that tumor promotion by iodine deficiency work through an increase of the serum thyroid-stimulating hormone (TSH) by negative feedback action that involves the pituitary-thyroid axis (Axelrad and Leblond, 1955; Ohshima and Ward, 1986). It has also been shown that lowered thyroid iodine content may sensitize the thyroid to TSH action (Bray, 1968) and that thyroid growth could be affected by iodide organification and altered production of the (growth-inhibiting) transforming growth factor-β (Yuasa et al. 1992). Iodine excess, on the other hand, is suggested to support tumorigenesis via a relationship to the escape from the Wolff-Chaicoff effect (Kanno et al. 1992) or by a mechanism acting on the serum hormone level (Nishikawa et al. 2005). Furthermore, iodine may affect development into a specific tumor type; iodine deficiency may promote follicular and anaplastic cancer, whereas in regions where there is sufficient iodine, papillary thyroid cancer is more predominant (Belfiore et al. 1987; Farahati et al. 2004). Thus, knowledge of iodine content and distribution in normal versus malignant thyroid tissue may be important to understanding the role of iodine in thyroid cancer development.

Thyroid iodine content (TIC) in normal thyroids is well documented, and several studies demonstrate large interindividual variation (Aeschimann et al. 1994; Fisher and Oddie, 1969; Milakovic et al. 2006; Zaichick and Choporov, 1996; Zaichick and Zaichick, 1997). However, information regarding iodine content in malignant thyroid tissue is scarce (Tadros et al. 1981). Two methods are available for investigating iodine content and distribution: X-ray fluorescence analysis (XRF) and secondary ion mass spectrometry (SIMS). XRF is a noninvasive method that can be used to quantify iodine and SIMS is a histological technique that can provide additional information regarding iodine distribution in the follicle structure (Fragu et al. 1989). With XRF, a sample is irradiated by photons. If the photon energy exceeds the binding energy of electrons in the innermost shells, emission of characteristic X-rays (with energies specific to the irradiated sample) will follow. Irradiating tissue that contains stable iodine will generate fluorescent radiation with intensity proportional to the amount of iodine in the irradiated sample. With SIMS, primary ions are sputtered on the sample surface, and the secondary ions are analyzed for composition and origin.

To perform accurate *in vitro* analysis, the original amount and distribution of iodine must be preserved; sample preparation is of paramount importance. For SIMS, Fragu et al. (Fragu et al. 1992) recommend chemical fixation with a mixture of glutaraldehyde and paraformaldehyde, i.e. Karnovsky fixative, followed by embedding in methacrylate. This method, which was evaluated for iodine loss by Rognoni et al. (Rognoni and Simon, 1974), has proven suitable for preservation of substances bound to macromolecules (like iodine bound to thyroglobulin [Tg]). XRF has been explored mostly for the possibility to be used *in vivo* but the method is also suitable for measuring iodine content *in vitro*. However, this application has not been widespread and the few published studies have prepared samples for XRF in different ways e.g. via destructive techniques such as lyophilization (freeze drying), homogenization (Aeschimann et al. 1994; Zaichick and Zaichick, 1997), or dissolving tissue in a sodium hydroxide solution (Tadros et al. 1981).

The first aim of the present study was to evaluate if freezing samples, which would facilitate XRF in clinical studies of small surgical tissue samples, is adequate for XRF analysis. The second aim was to use XRF to evaluate the efficacy and benefits of using Karnovsky fixative in place of other aldehydes in sample preparation for SIMS analysis.

## Materials and Methods

Ten porcine thyroid glands were collected at the local slaughterhouse. Porcine thyroids were used because of the homogeneous material. The pigs came from the same farm and had been fed the same diet. After excision from the newly slaughtered animals, the glands were immediately cooled with ice. Each thyroid was cut into four pieces giving 40 pieces altogether, which weighed 0.21 g on average, varying between 0.09–0.45 g. Within 24 hours, all pieces were measured with XRF twice for 5 minutes. These values represented the baseline values. The pieces were then assigned to one of four preparation groups: freezing or fixation in one of three aldehydes for investigation of the sample preparation methods.

### Sample preparation

**Figure 1:**
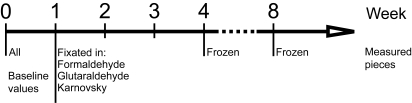
Time axis for the experimental set-up. The samples fixated in formaldehyde, glutaraldehyde and Karnovsky were measured 1 week after that the baseline values were obtained whereas the ten frozen samples were analyzed 4 and 8 weeks after the baseline values.

One piece from each of the ten thyroids was stored in a plastic container with an air-sealed cap at −20 °C. These ten frozen pieces were not further divided or treated in any other way before analysis of iodine content with XRF after 4 and 8 weeks (Fig. 1). During the measurement after 4 weeks the pieces started to thaw but were freezed again directly after the analysis and remained as such until the final measurement after 8 weeks. The three remaining pieces from each gland were, directly after baseline measurements, fixated in different aldehydes: formaldehyde (2%), glutaraldehyde (2.5%), and a modified Karnovsky fixative (2% paraformaldehyde and 2.5% glutaraldehyde in 0.05 M sodium cacodylate buffer, pH 7.2). These pieces were measured with XRF 1 week after the baseline values were obtained. All results were compared to the baseline values.

### XRF analysis

**Figure 2:**
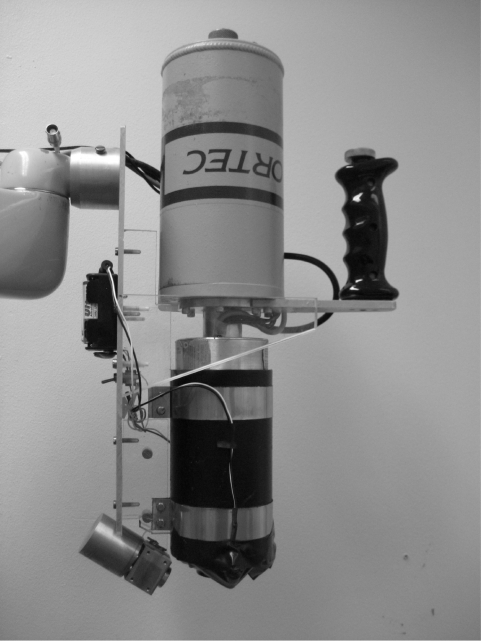
XRF system used for analysis of the tissue samples. The angle between the source and detector was 56° and the source collimator aperture was 8 mm, thus giving a radiation field having greater extension than the sample dimensions.

To detect the characteristic emitted X-rays, a XRF system with ^241^Am (11.1 GBq) as the radiation source and a planar HPGe detector (Fig. 2) (EG&G; ORTEC, Oak Ridge, Tennessee, USA; Ø = 25 mm, thickness 10 mm) was used (Hansson et al. 2004). The system was calibrated with iodine solutions of different concentrations; the volumes were within the same order of magnitude as the sample volumes. Calibrations and measurements were performed in the same geometry; the samples were placed on an air-equivalent surface, positioned at the intersection of the source and detector collimator openings. Each sample was measured twice for 5 minutes. The measurement and positioning uncertainty for the system was 7%.

### Statistical methods

For investigation of the iodine change (compared to the baseline values) due to preservation by fixation in formaldehyde, glutaraldehyde or Karnovsky or by freezing, paired two-sample t-tests of the mean were performed. Two-sample t-tests of mean were used to see if Karnovsky fixative was superior to formaldehyde or glutaraldehyde.

## Results

The mean iodine concentration in the 10 porcine thyroid glands was 1.08 mg/ml (SD 0.26 mg/ml), obtained from the baseline measurements. Iodine content generally varied little between the four different pieces from the same thyroid gland; the standard deviation was 0.11 mg/ml.

There were no significant changes in iodine content due to freezing (Table 1). Freezing for 4 weeks produced no more than a 10% change in the iodine content; for one gland the value increased to 24% after 8 weeks of freezing. For all the samples fixed in an aldehyde there was a loss of iodine (Table 2). The decrease in iodine content from baseline was significant for samples fixed in either aldehyde (p < 0.05). Karnovsky was the best fixative in this regard, yielding a mean 14% loss compared to 20% and 30% for glutaraldehyde and formaldehyde, respectively. Fixation with Karnovsky fixative yielded significantly (p < 0.05) lower iodine loss than fixation with formaldehyde, but not glutaraldehyde.

## Discussion

XRF and SIMS are powerful methods for *in vitro* quantification and histological mapping of iodine in thyroid tissue. If samples are not analyzed directly upon excision, the tissue must be preserved in a way that minimizes potential loss of iodine. For XRF, there is no standard procedure for sample preparation; the methods used vary (Tadros et al. 1981; Zaichick and Zaichick, 1997). However, for SIMS, the tissue structure is preserved by a series of steps that include fixation in an aldehyde, a method that has been evaluated with respect to iodine loss (Rognoni and Simon, 1974).

It was found that the samples could be stored at −20 °C for 2 months without loss of iodine content. This result is consistent with previous investigations showing no TIC loss due to lyophilized drying (Zaichick and Choporov, 1996). For most of the samples, the iodine signal after 1 month was slightly higher. This can be accounted for by a decrease in sample volume due to the time-dependent evaporation of water from the tissue. When measuring the low fluorescent energies that are associated with iodine (28.6 keV), a small decrease in volume can greatly affect photon attenuation (Hansson et al. 2007). A smaller volume means less material to be penetrated (i.e. to be absorbed by or scattered in) by the incoming radiation from ^241^Am as well as the emitted characteristic X-rays, and will thus lead to a larger signal. A calculation for each sample how much thinner the thyroid sections would need to be to cause the observed increment in iodine signal, resulted in a mean value of 0.7 mm. It should also be remembered that the results include a measurement and positioning uncertainty, which is 7% for the XRF system used in this study. As the results from freezing the samples for 4 weeks, the results after 8 weeks presented non significant changes in iodine content compared to the baseline values. The changes were in the same range as of measurement and positioning uncertainty.

Sample size may affect measurements in other ways as well. First, to avoid misleading results for different sized samples with an equal concentration of iodine, the samples should either be well within the analyzed volume (the volume covered by both the irradiation and the detection views) or much larger than the analyzed volume (Hansson et al. 2004). Second, iodine is not homogeneously distributed within the thyroid. Differences may exist between different parts of the gland (Zaichick and Choporov, 1996) and, on a smaller scale, between adjacent follicles (Fragu et al. 1989; Robison and Davis, 1969), although this seems to be more rare (Lowenstein and Wollman, 1967; Robison and Davis, 1969). Differences between different parts of the gland were observed even in the present study, where the thyroid sections were small. The mean difference between the highest and lowest measured iodine concentration for the same gland (averaged over 10 glands) was 0.24 mg/ml. Thus, absolute comparison of TIC between glands requires the whole gland. Homogenization and dissolving the thyroid ensure that the iodine concentration within the solution represents the mean for the entire gland. However, a drawback with this approach is the possibility for a large amount of iodine to be lost during homogenization. Freezing the untreated sample preserves the gross structures in the thyroid. When the purpose is to study surgical samples (which are generally small), freezing at −20 °C, which is easier than both homogenization and lyophilization, is more attractive. We believe that if exceptionally low or high iodine content can promote the development of follicular or papillary thyroid cancer, respectively, the iodine content in smaller samples yields more information than does analysis of the entire gland. In that case, it might be useful to examine pieces from different parts of the gland, malignant as well as benign, with the purpose of obtaining information about glandular iodine distribution.

Samples for SIMS analysis are fixed in an aldehyde, embedded in methacrylate, and cut into μm slices. This is a necessary procedure, and iodine loss during these steps is difficult to avoid. The study results suggest that Karnovsky fixative is superior to formaldehyde and glutaraldehyde alone (14% iodine loss compared to 30% and 20%, respectively). This was expected based on the fixatives’ chemical properties. Formaldehyde has one aldehyde group and rapidly penetrates tissues, but its reactions with proteins, cross-linking to form methylene bridges, occur slowly. Glutaraldehyde has two aldehyde groups that are separated by a chain of three methylene bridges, thus increasing the possibility of cross-linking relative to formaldehyde. Glutaraldehyde rapidly reacts with proteins, but the larger molecules (compared to formaldehyde) penetrate tissue more slowly (Kiernan, 2000). Karnovsky fixative, being a mixture of formaldehyde and glutaraldehyde, combines rapid tissue penetration by formaldehyde initiating structural stabilization, with fast reaction with proteins and more thorough tissue stabilization by the slowly penetrating glutaraldehyde. Thus, Karnovsky seems to be a good fixative that should preserve the structural integrity and chemical composition of tissue.

In normal thyroids, approximately 83% of the total amount of iodine is bound to Tg (Aeschimann et al. 1994). In many cases, bound substances, such as iodine bound to Tg, are insensitive to the preservation method and only mobile ions are affected (Robison and Davis, 1969). This implies that analysis of TIC might be relatively stable with respect to pretreatment methods. Rognoni et al. found that solubilization in 2% glutaraldehyde produced a 5% loss of iodine, most of which was in the form of iodo-proteins. As much as 20% of the total iodine content was lost when sectioning thyroid samples; this was presumed to be due to scraping of colloid. In the present study, sectioning was performed before immersing the samples in 2.5% glutaraldehyde. Sectioning leaves a surface with an open follicle structure, from which iodine can be lost. Direct freezing may prevent this escape, but when immersing the sample in, for example, an aldehyde, the injured follicles may be emptied of their remaining iodine content. The observed 20% decrease in iodine registrations when fixing in glutaraldehyde may thus reflect the escape of scraped colloid as well as true solubilization in the aldehyde. In that case, the iodine depletion observed in the present study can be considered to be within the same order of magnitude as that found by Rognoni et al. Such losses are not negligible and are important to be aware of since they could lead to appreciable underestimations of iodine content. For XRF, iodine losses may be minimized simply by avoiding sectioning, which is the most critical point of iodine loss. Therefore, the use of preservation methods involving homogenization, which leads to exposure of large tissue areas from which iodine can escape, could be substituted by freezing.

The iodine concentrations in benign and malignant thyroid tissue may differ (Tadros et al. 1981). How and to what extent this is true can be studied using XRF and SIMS. Knowledge of the iodine content and distribution in a tumor and in the tissue it grows from might provide additional knowledge regarding how iodine intake and storage within the thyroid affects the development into different tumor types. It is therefore of major importance that we use all available methods to gain knowledge concerning the role of TIC in both thyroid health and disease. When doing so, it is imperative that the sample preparation methods are used correctly and that possible methodological shortcomings are recognized, so that the result will be as accurate as possible.

## Conclusion

The choice of sample preservation method influences TIC. Freezing of untreated tissue for 1 or 2 months, the suggested preservation method for XRF analysis, yielded no significant iodine loss, whereas fixation with aldehydes yielded significant iodine loss after 1 week. Karnovsky fixative proved to be the best fixative. Therefore, SIMS analysis should be performed with samples fixed with Karnovsky fixative, and XRF analysis should be performed with untreated frozen tissue. To our knowledge, this simple preservation method for XRF analysis has not previously been investigated. We believe that it will be valuable for studies of iodine content in small benign and malignant tissue samples collected at surgery.

## Figures and Tables

**Table 1:** The volume, baseline iodine content and concentration for the 10 tissue pieces that were frozen together with the iodine content and iodine loss in %, compared to the baseline values, due to freezing for 4 and 8 weeks.

			Baseline values		4 weeks		8 weeks	
Thyroid	Piece	Volume (ml)	Iodine content (mg)	Iodine concentration (mg/ml)	Iodine content (mg)	Iodine loss %	Iodine content (mg)	Iodine loss %
1	4	0.273	0.28	1.01	0.30	−9	0.25	8
2	8	0.244	0.23	0.92	0.23	−3	0.25	−10
3	12	0.374	0.42	1.14	0.43	0	0.40	6
4	16	0.139	0.16	1.17	0.18	−10	0.16	0
5	20	0.113	0.15	1.31	0.15	−2	0.14	4
6	24	0.240	0.29	1.22	0.30	−3	0.27	7
7	28	0.097	0.13	1.32	0.11	10	0.10	24
8	32	0.086	0.11	1.25	0.11	0	0.10	4
9	36	0.109	0.15	1.34	0.16	−9	0.16	−8
10	40	0.156	0.06	0.42	0.07	−2	0.08	6

**Table 2:** Volume, baseline iodine content and concentration for the 30 tissue pieces that were fixated either in formaldehyde, glutaraldehyde or Karnovsky fixative. The iodine content and iodine loss in %, compared to the baseline values, due to fixation is reported.

			Baseline values			I week	
Thyroid	Piece	Volume (ml)	Iodine content (mg)	Iodine concentration (mg/ml)	Fixation	Iodine content (mg)	Iodine loss %
1	1	0.259	0.26	1.01	Formaldehyde	0.13	52
	2	0.200	0.20	1.01	Glutaraldehyde	0.18	9
	3	0.229	0.27	1.16	Karnovsky	0.19	29
2	5	0.231	0.23	1.01	Formaldehyde	0.17	28
	6	0.289	0.28	0.96	Glutaraldehyde	0.21	23
	7	0.340	0.30	0.89	Karnovsky	0.23	23
3	9	0.277	0.34	1.24	Formaldehyde	0.24	30
	10	0.451	0.48	1.07	Glutaraldehyde	0.31	37
	11	0.319	0.39	1.21	Karnovsky	0.35	10
4	13	0.165	0.20	1.20	Formaldehyde	0.15	23
	14	0.162	0.21	1.31	Glutaraldehyde	0.19	12
	15	0.167	0.22	1.33	Karnovsky	0.18	17
5	17	0.119	0.18	1.51	Formaldehyde	0.13	28
	18	0.161	0.22	1.38	Glutaraldehyde	0.20	11
	19	0.139	0.20	1.47	Karnovsky	0.20	2
6	21	0.196	0.29	1.50	Formaldehyde	0.23	20
	22	0.237	0.28	1.20	Glutaraldehyde	0.24	16
	23	0.216	0.24	1.12	Karnovsky	0.21	11
7	25	0.212	0.14	0.65	Formaldehyde	0.09	31
	26	0.176	0.12	0.71	Glutaraldehyde	0.10	20
	27	0.214	0.15	0.70	Karnovsky	0.13	11
8	29	0.143	0.16	1.13	Formaldehyde	0.12	25
	30	0.137	0.15	1.10	Glutaraldehyde	0.12	22
	31	0.218	0.22	1.01	Karnovsky	0.21	4
9	33	0.149	0.20	1.32	Formaldehyde	0.16	18
	34	0.185	0.21	1.14	Glutaraldehyde	0.16	22
	35	0.191	0.23	1.18	Karnovsky	0.18	22
10	37	0.203	0.10	0.51	Formaldehyde	0.06	44
	38	0.226	0.13	0.57	Glutaraldehyde	0.10	25
	39	0.188	0.09	0.48	Karnovsky	0.08	8
